# Supplementary figures and images for: Variable generalization performance of a deep learning model to detect pneumonia in chest radiographs: A cross-sectional study
Source: PLoS Med. 2018 Nov 6;15(11):e1002683. doi: 10.1371/journal.pmed.1002683 (PMC6219764; doi:10.1371/journal.pmed.1002683)

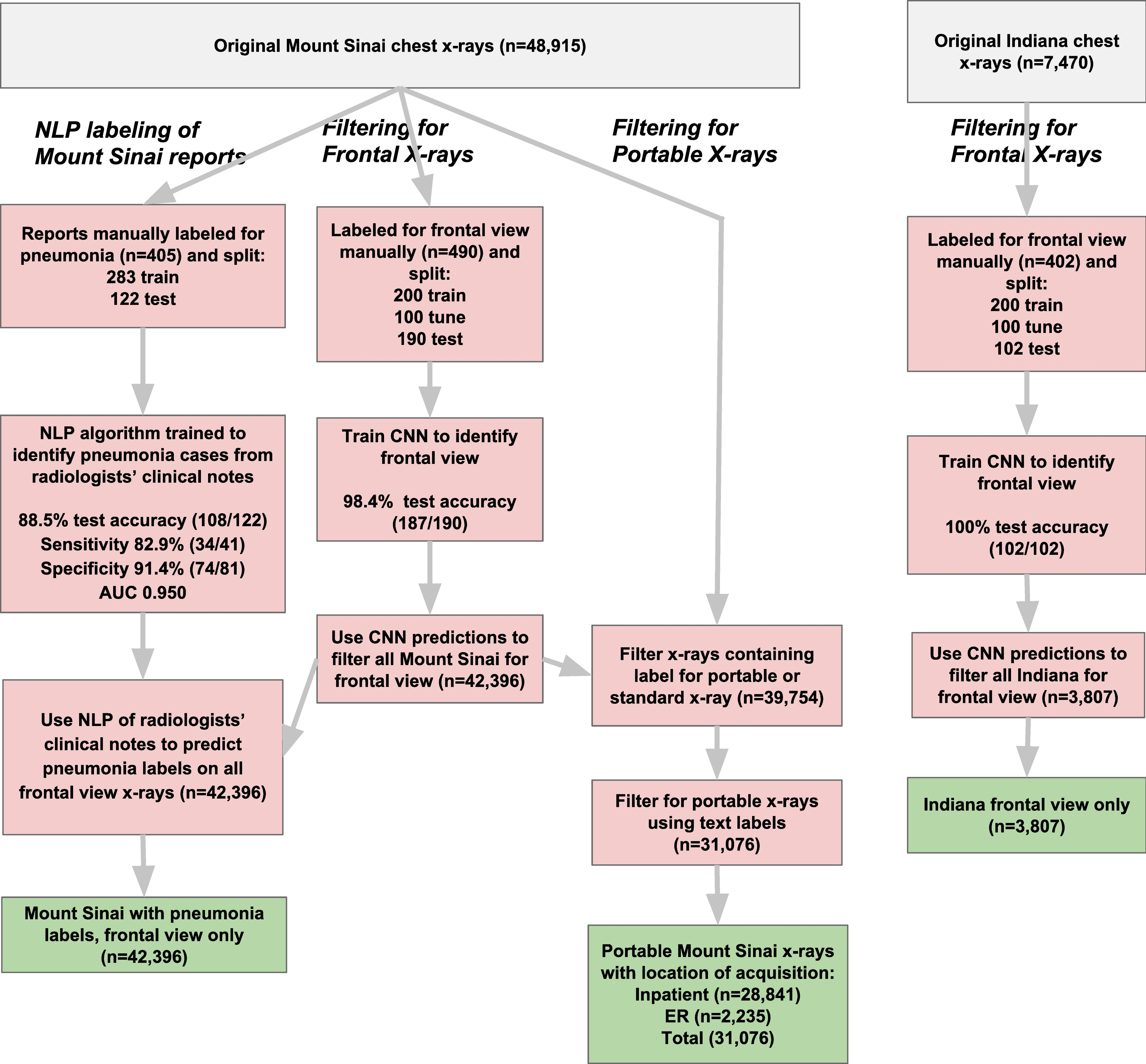

Supplement: S1 Fig — (TIF) [file pmed.1002683.s003.tif]

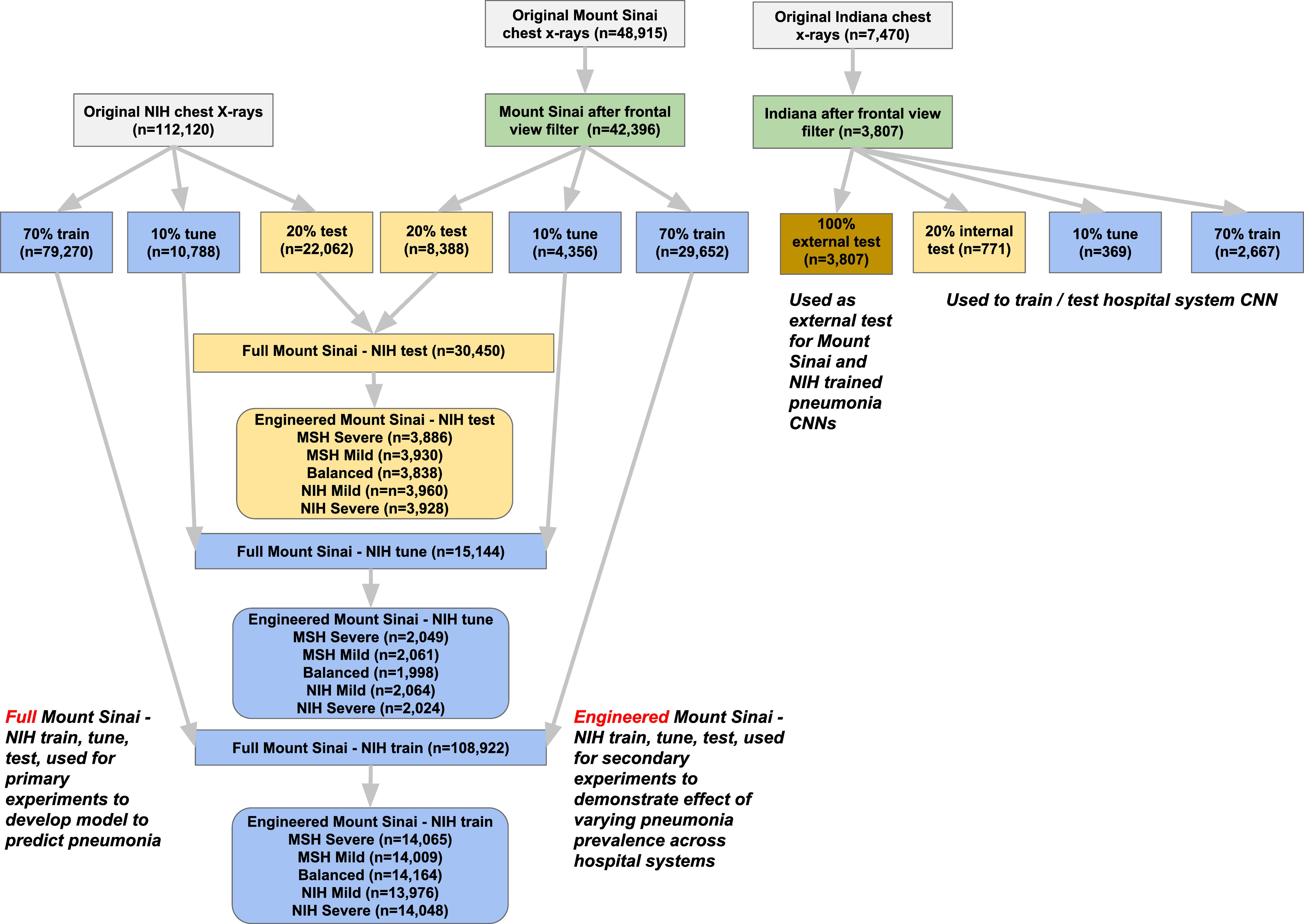

Supplement: S2 Fig — (TIF) [file pmed.1002683.s004.tif]

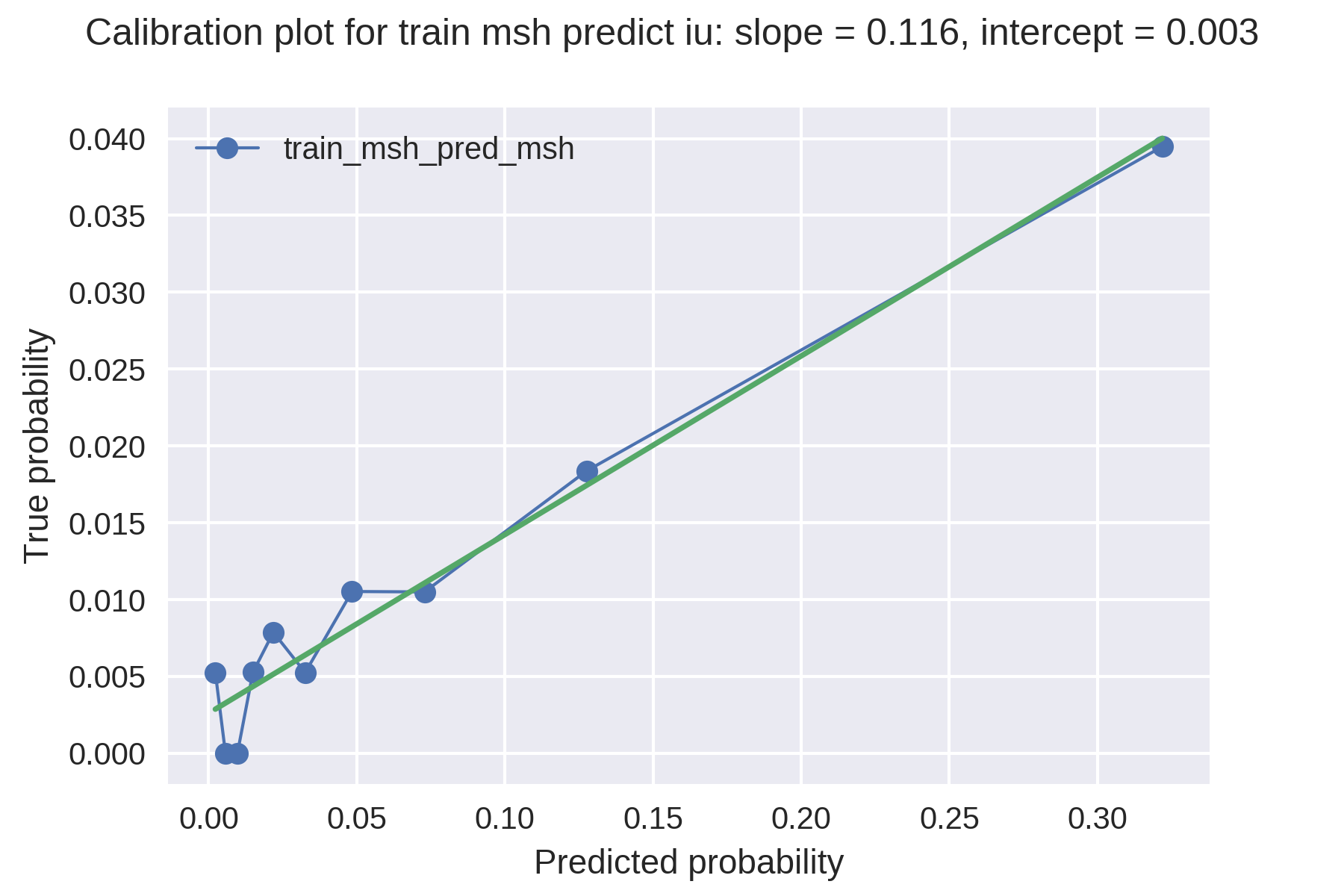

Supplement: S3 Fig — IU, Indiana University Network for Patient Care; MSH, Mount Sinai Hospital. (TIF) [file pmed.1002683.s005.tif]

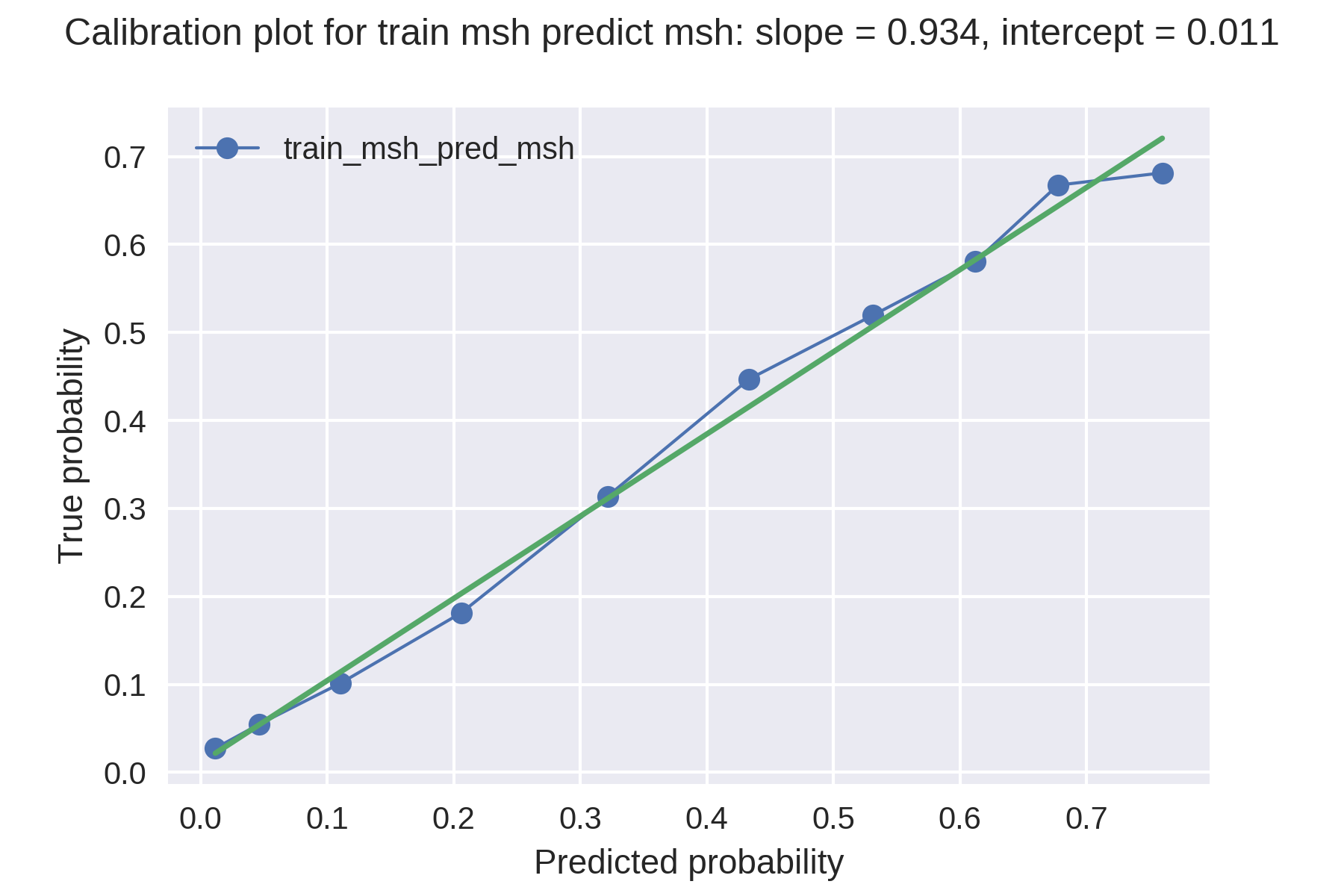

Supplement: S4 Fig — MSH, Mount Sinai Hospital. (TIF) [file pmed.1002683.s006.tif]

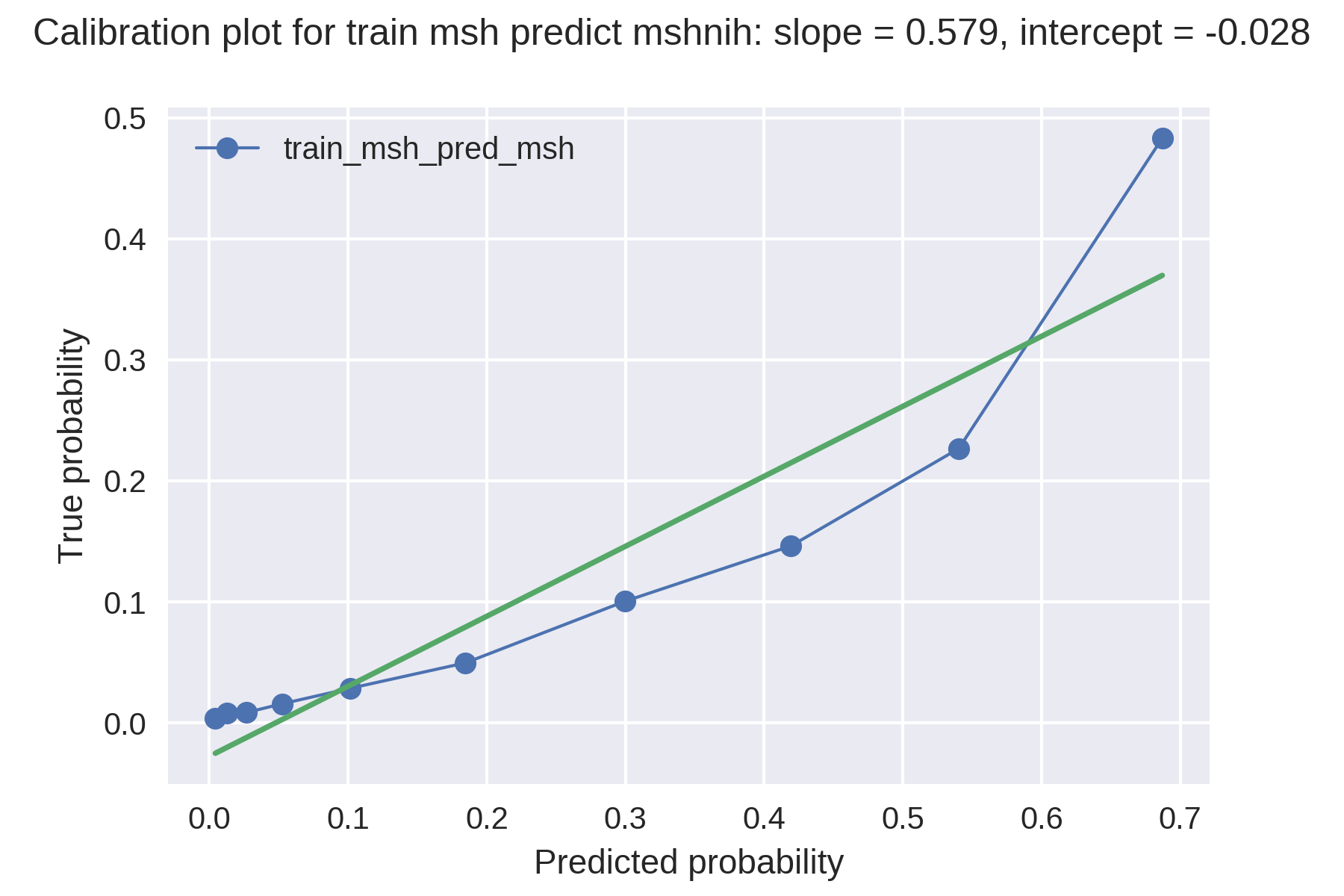

Supplement: S5 Fig — MSH, Mount Sinai Hospital; NIH, National Institutes of Health Clinical Center. (TIF) [file pmed.1002683.s007.tif]

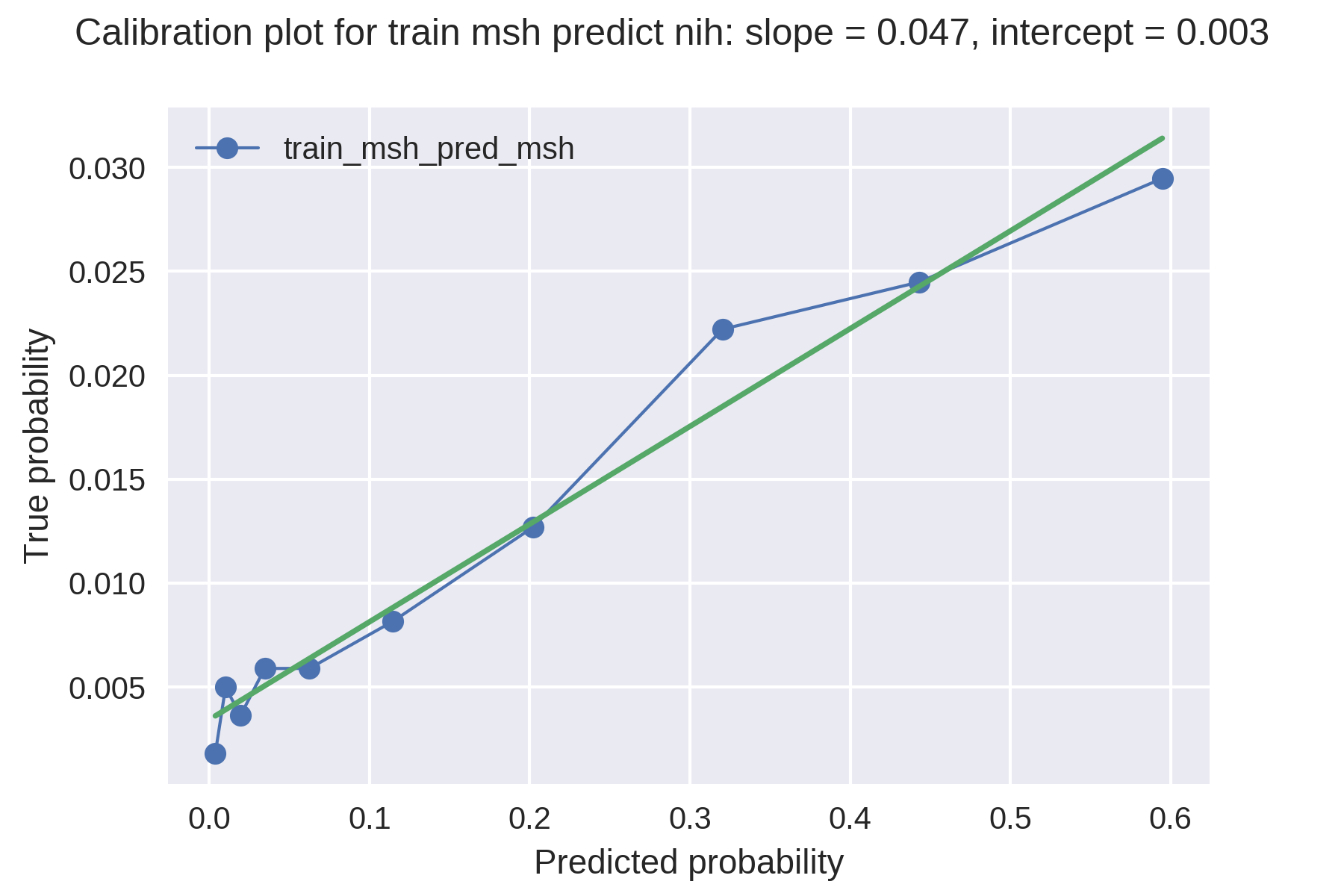

Supplement: S6 Fig — MSH, Mount Sinai Hospital; NIH, National Institutes of Health Clinical Center. (TIF) [file pmed.1002683.s008.tif]

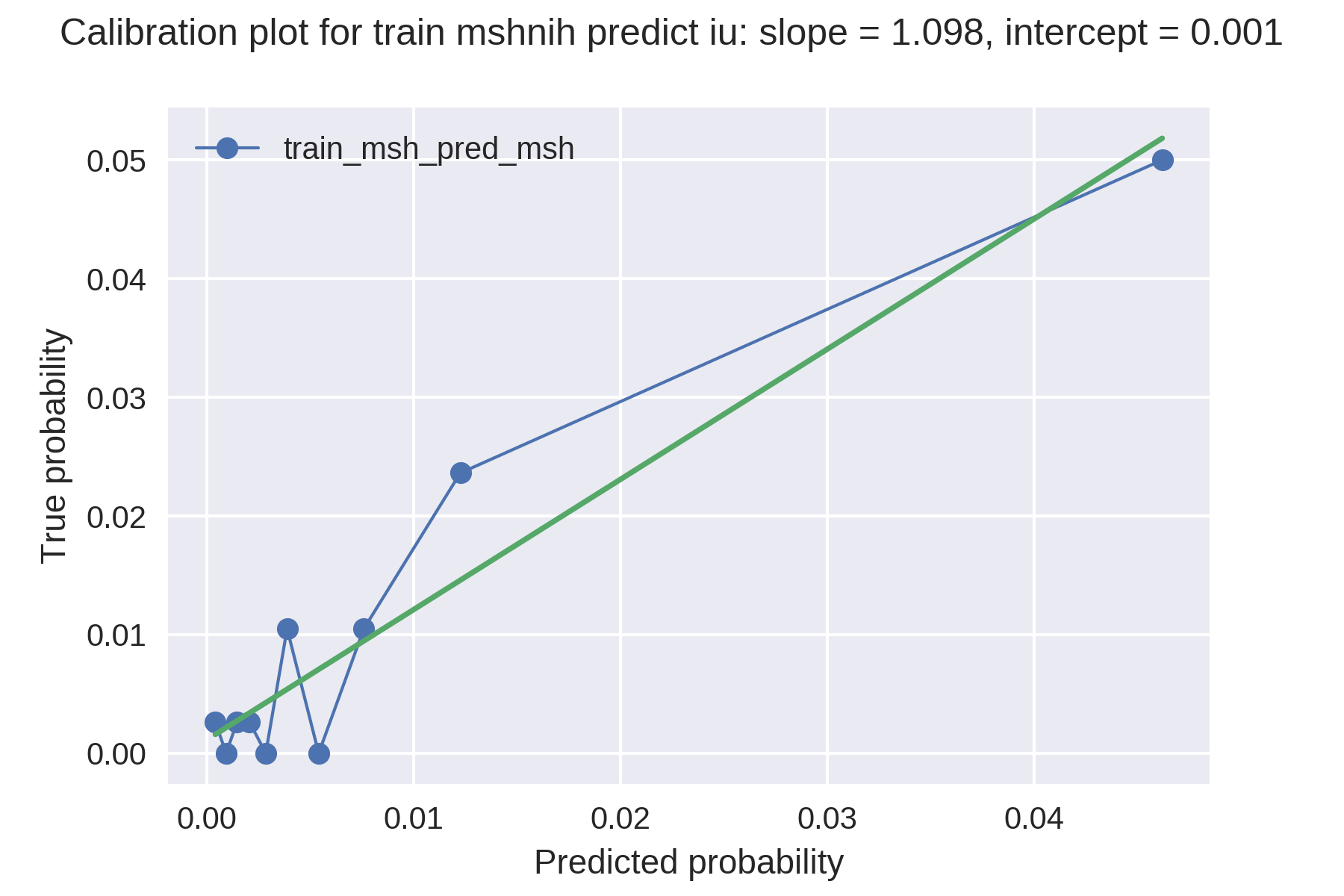

Supplement: S7 Fig — IU, Indiana University Network for Patient Care; MSH, Mount Sinai Hospital; NIH, National Institutes of Health Clinical Center. (TIF) [file pmed.1002683.s009.tif]

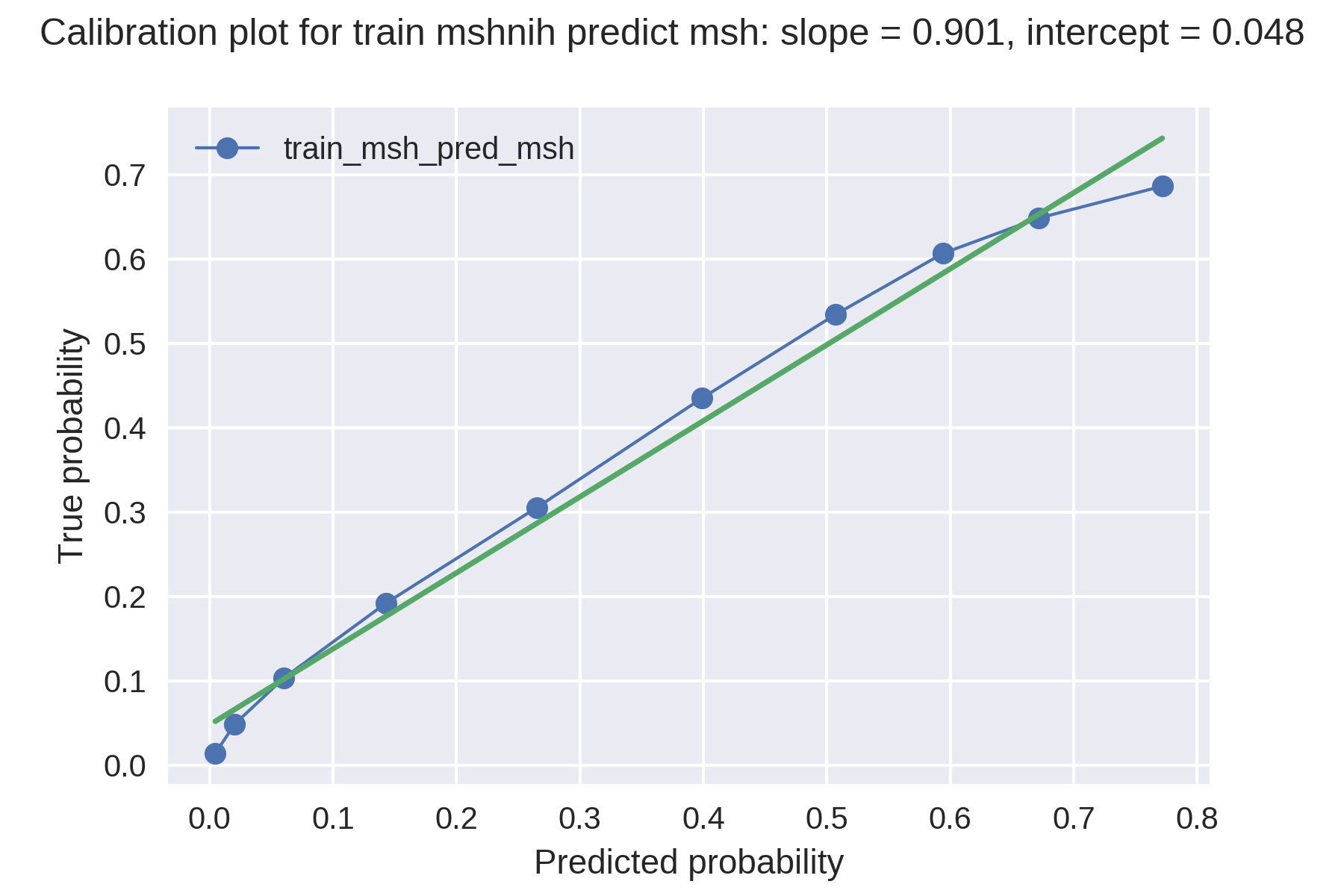

Supplement: S8 Fig — MSH, Mount Sinai Hospital; NIH, National Institutes of Health Clinical Center. (TIF) [file pmed.1002683.s010.tif]

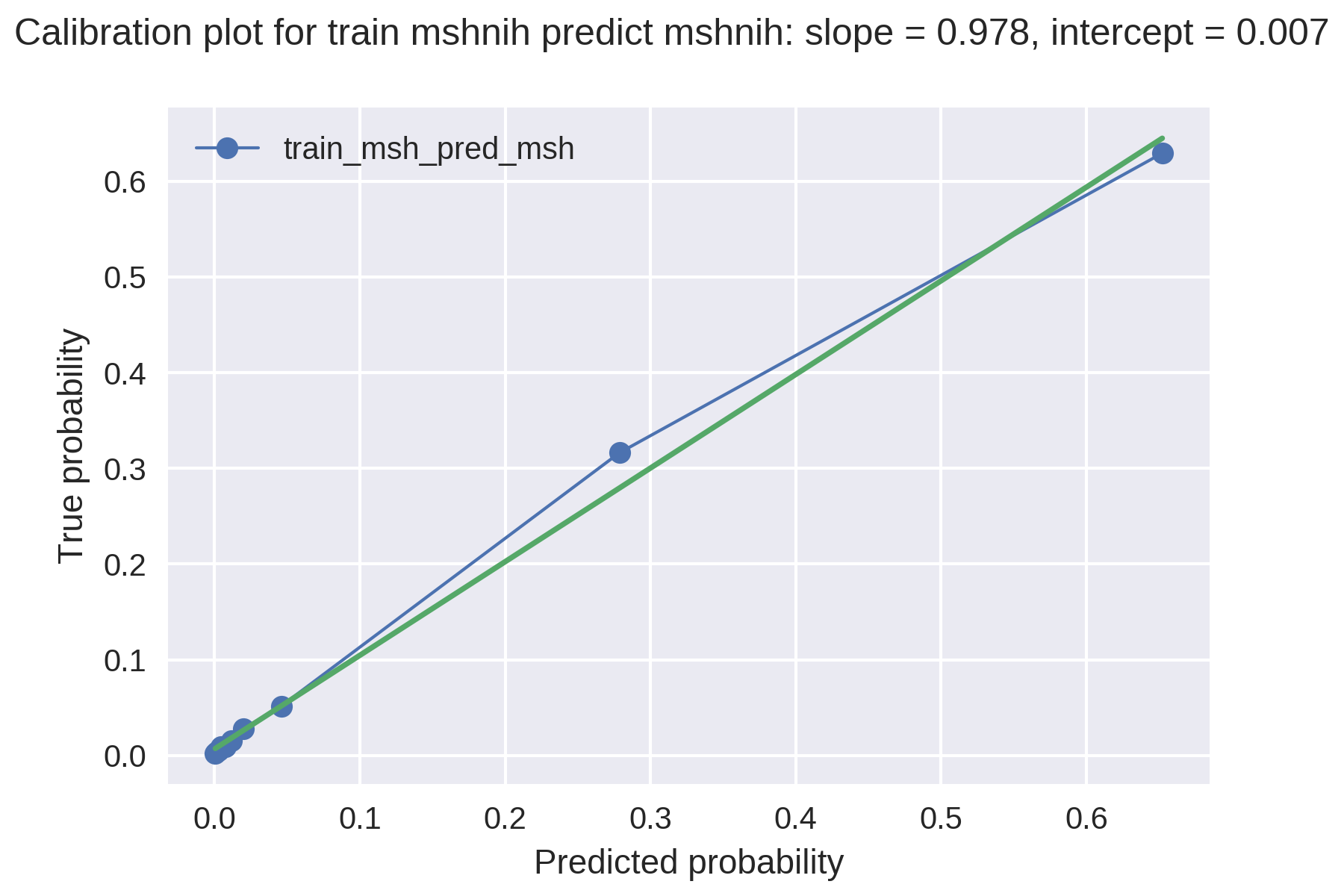

Supplement: S9 Fig — MSH, Mount Sinai Hospital; NIH, National Institutes of Health Clinical Center. (TIF) [file pmed.1002683.s011.tif]

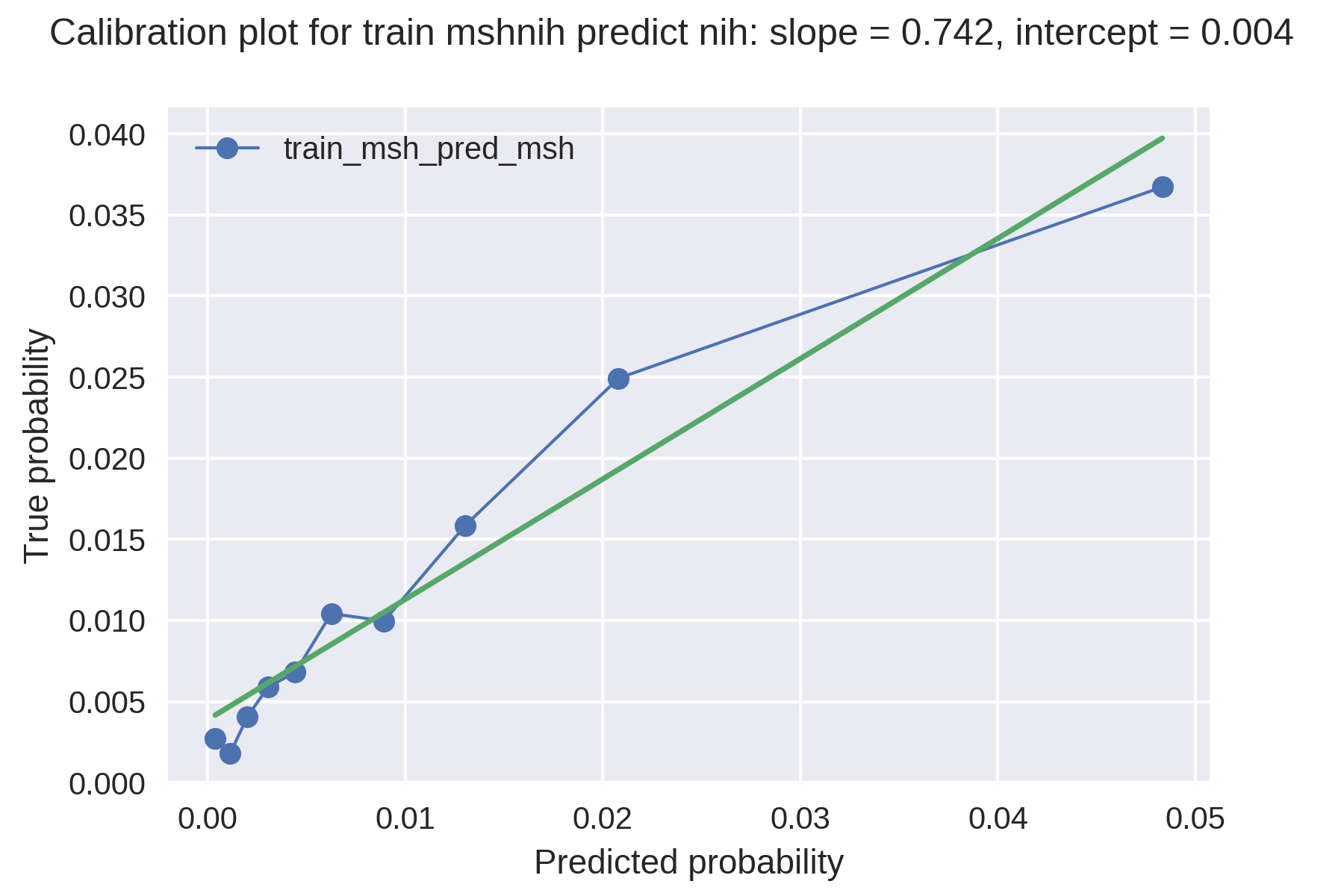

Supplement: S10 Fig — MSH, Mount Sinai Hospital; NIH, National Institutes of Health Clinical Center. (TIF) [file pmed.1002683.s012.tif]

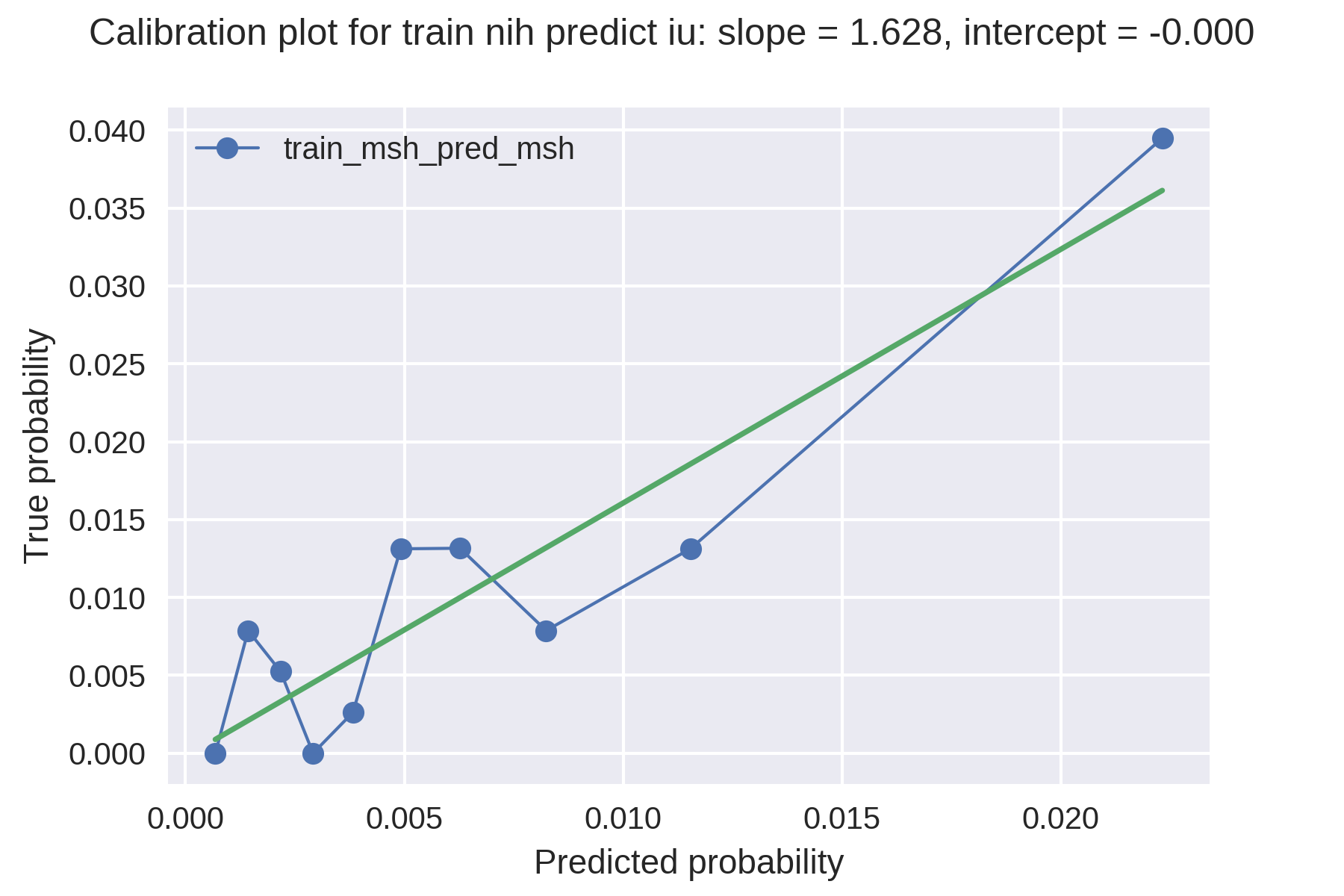

Supplement: S11 Fig — IU, Indiana University Network for Patient Care; NIH, National Institutes of Health Clinical Center. (TIF) [file pmed.1002683.s013.tif]

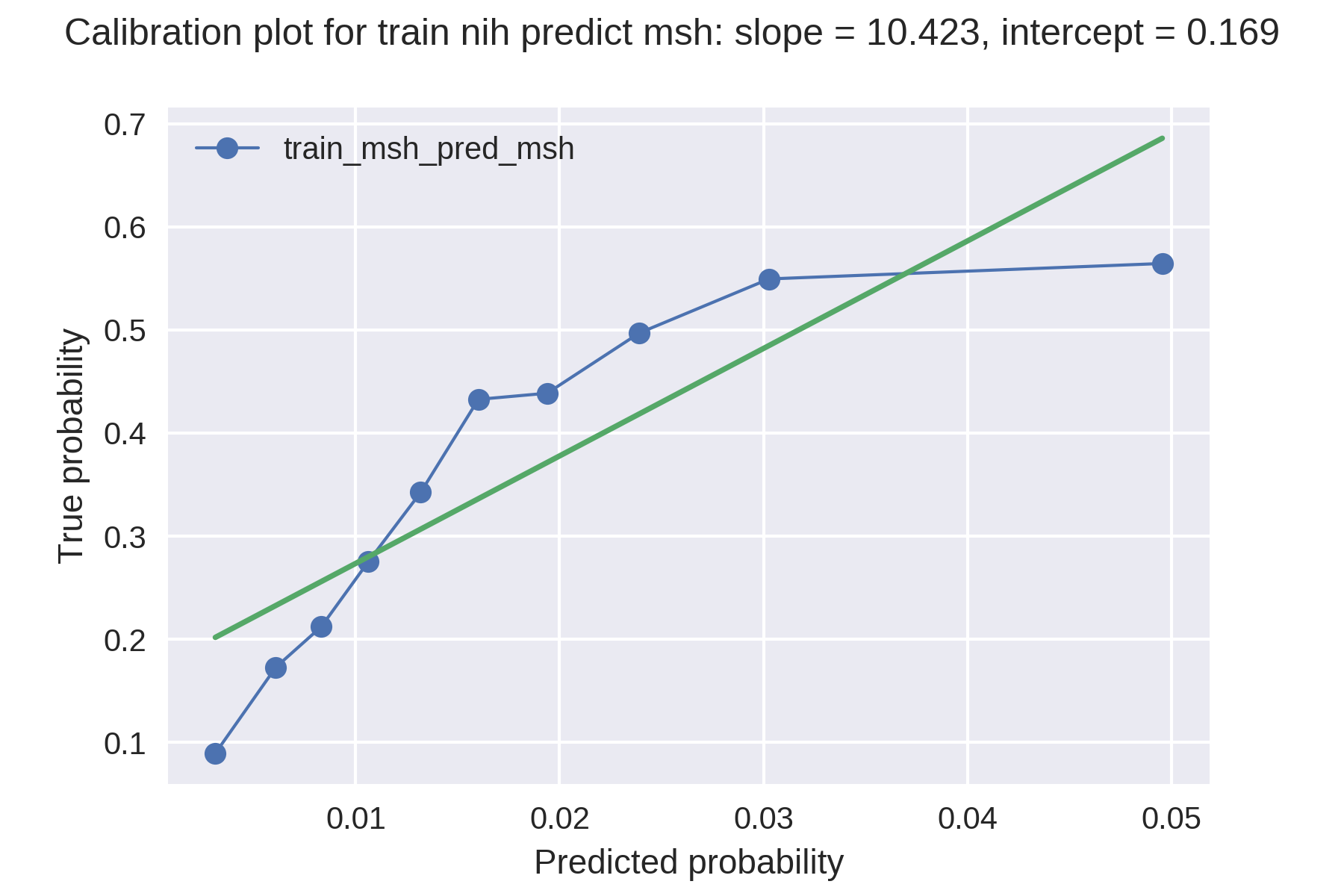

Supplement: S12 Fig — MSH, Mount Sinai Hospital; NIH, National Institutes of Health Clinical Center. (TIF) [file pmed.1002683.s014.tif]

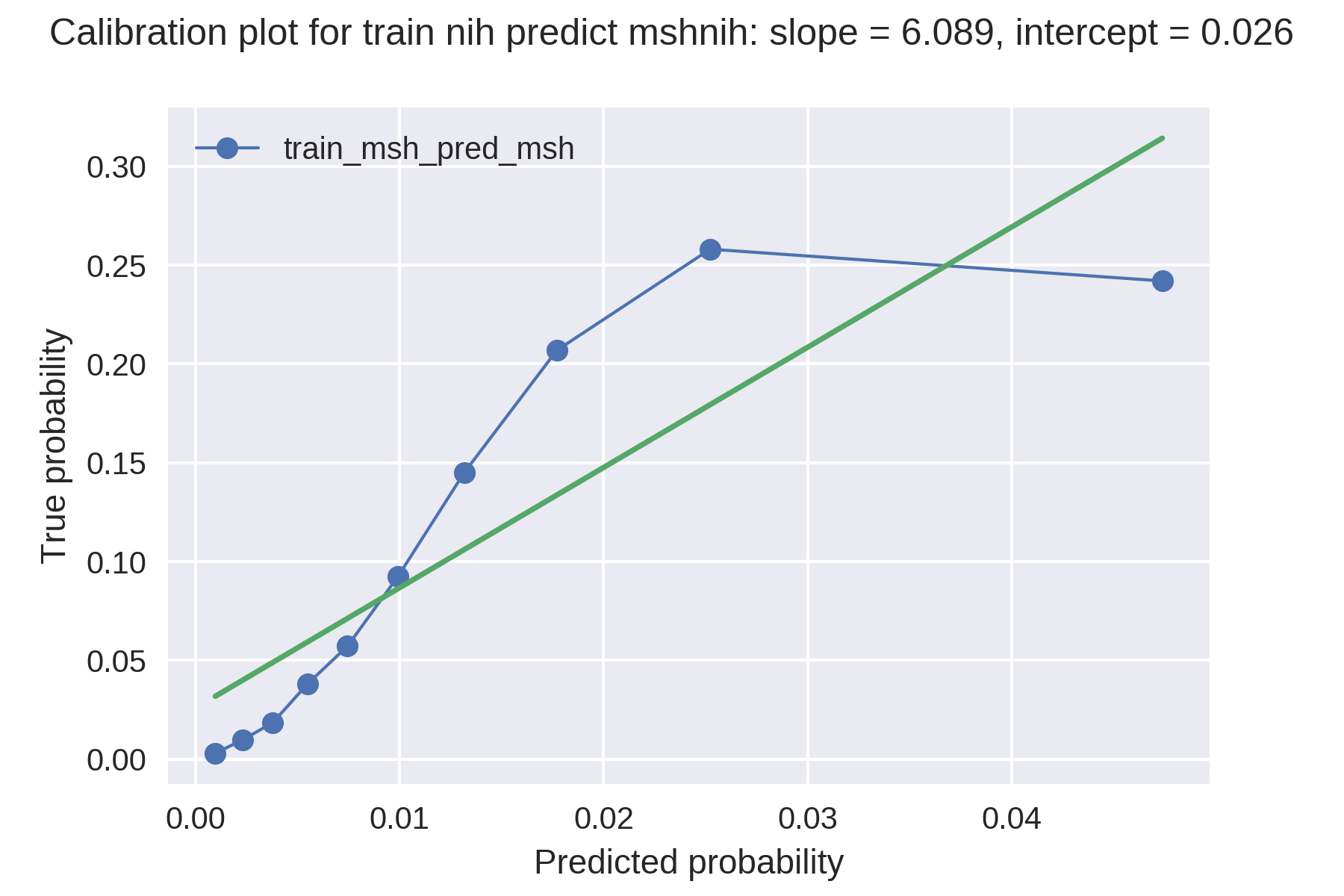

Supplement: S13 Fig — MSH, Mount Sinai Hospital; NIH, National Institutes of Health Clinical Center. (TIF) [file pmed.1002683.s015.tif]

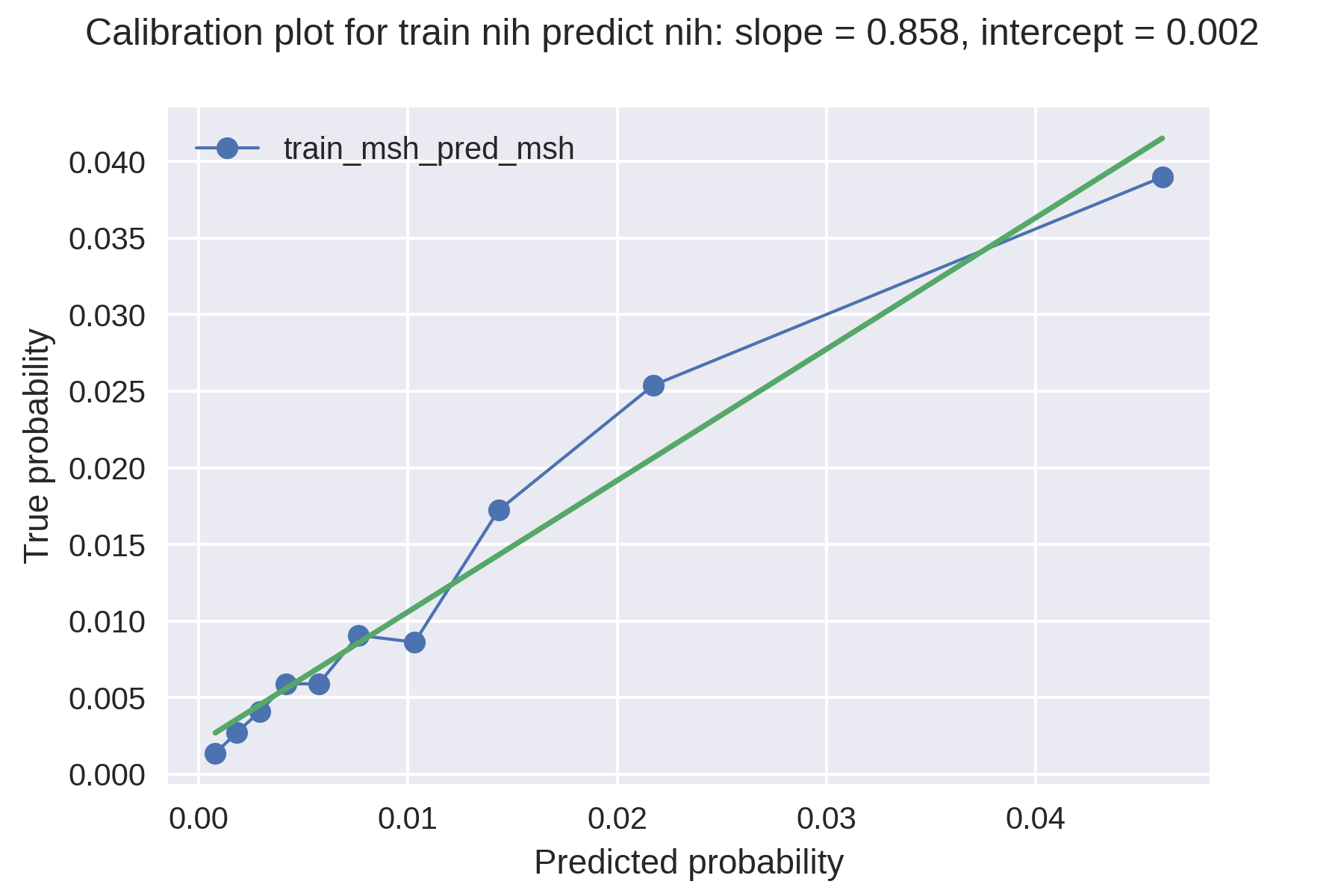

Supplement: S14 Fig — NIH, National Institutes of Health Clinical Center. (TIF) [file pmed.1002683.s016.tif]

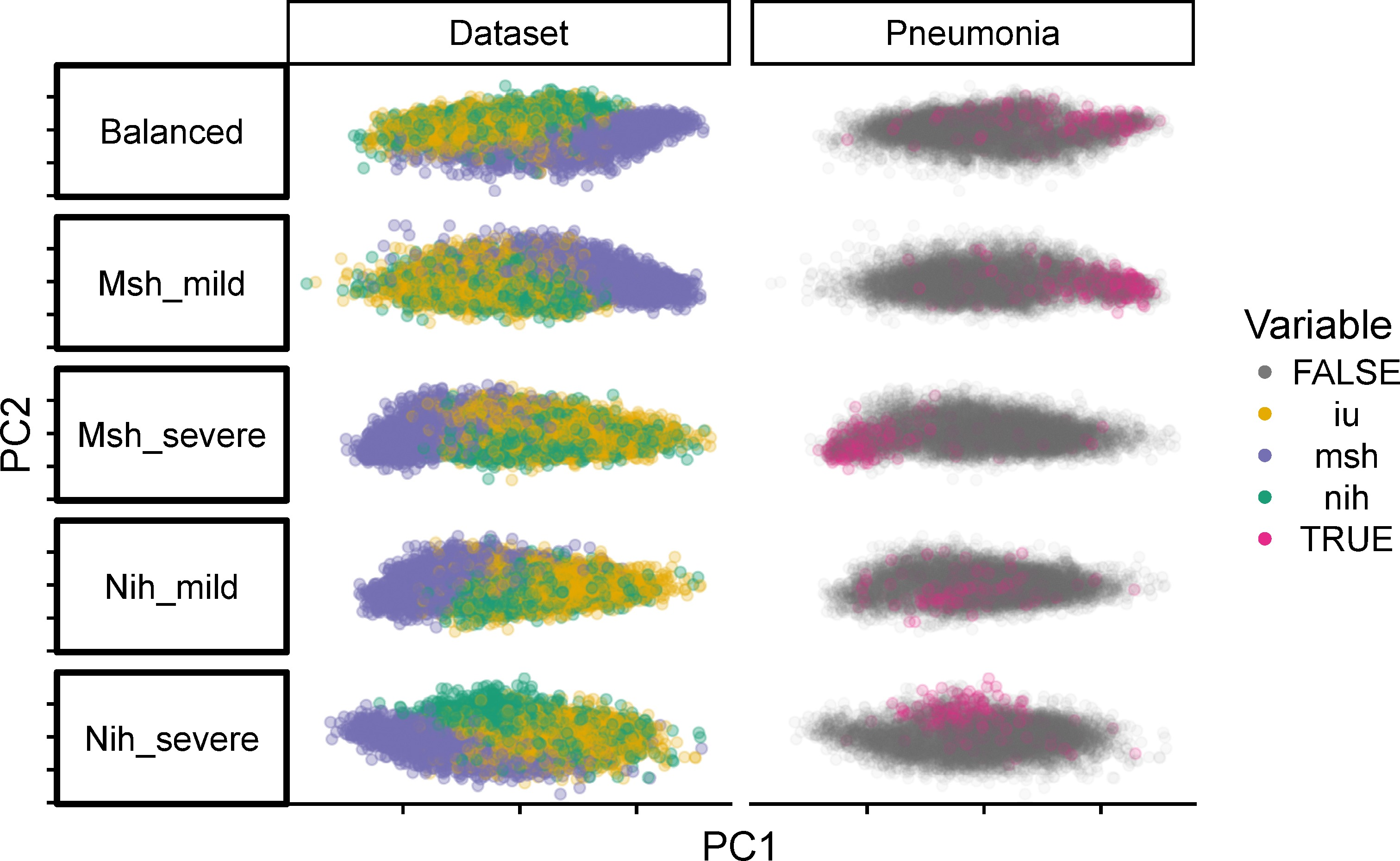

Supplement: S15 Fig — Principal component analysis was performed on each image’s bottleneck features and colored to reveal the distribution of hospital systems and pneumonia. MSH images are largely separable from radiographs acquired at IU and NIH. IU, Indiana University Network for Patient Care; MSH, Mount Sinai Hospital; NIH, National Institutes of Health Clinical Center. (TIF) [file pmed.1002683.s017.tif]
